# Treatment temperature and insult severity influence the neuroprotective effects of therapeutic hypothermia

**DOI:** 10.1038/srep23430

**Published:** 2016-03-21

**Authors:** Thomas Wood, Damjan Osredkar, Maja Puchades, Elke Maes, Mari Falck, Torun Flatebø, Lars Walløe, Hemmen Sabir, Marianne Thoresen

**Affiliations:** 1 Department of Physiology, Institute of Basic Medical Sciences, University of Oslo, Oslo, Norway; 2 Department of Paediatric Neurology, University Children’s Hospital, Ljubljana, Slovenia; 3 Department of General Pediatrics, Neonatology and Pediatric Cardiology, University Children’s Hospital, Heinrich-Heine University, Düsseldorf, Germany; 4 Neonatal Neuroscience, School of Clinical Sciences, University of Bristol, Bristol, United Kingdom

## Abstract

Therapeutic hypothermia (HT) is standard care for moderate and severe neonatal hypoxic-ischaemic encephalopathy (HIE), the leading cause of permanent brain injury in term newborns. However, the optimal temperature for HT is still unknown, and few preclinical studies have compared multiple HT treatment temperatures. Additionally, HT may not benefit infants with severe encephalopathy. In a neonatal rat model of unilateral hypoxia-ischaemia (HI), the effect of five different HT temperatures was investigated after either moderate or severe injury. At postnatal-day seven, rat pups underwent moderate or severe HI followed by 5 h at normothermia (37 °C), or one of five HT temperatures: 33.5 °C, 32 °C, 30 °C, 26 °C, and 18 °C. One week after treatment, neuropathological analysis of hemispheric and hippocampal area loss, and CA1 hippocampal pyramidal neuron count, was performed. After moderate injury, a significant reduction in hemispheric and hippocampal loss on the injured side, and preservation of CA1 pyramidal neurons, was seen in the 33.5 °C, 32 °C, and 30 °C groups. Cooling below 33.5 °C did not provide additional neuroprotection. Regardless of treatment temperature, HT was not neuroprotective in the severe HI model. Based on these findings, and previous experience translating preclinical studies into clinical application, we propose that milder cooling should be considered for future clinical trials.

Perinatal asphyxia currently affects 3–5 out of every 1,000 live term births, with around 20% of those experiencing moderate or severe encephalopathy of hypoxic-ischaemic origin (HIE)^1^. For these infants, therapeutic hypothermia (HT) (cooling to 33.5–34.5 °C for 72 h) is the current standard of care^2^. As HT becomes a mainstream therapy worldwide, questions have arisen regarding whether the current cooling protocols provide optimal neuroprotection, or may even be harmful in the setting of certain comorbidities^3^,^4^. In order to further develop cooling protocols for neonates, more direct experimental comparison of a range of temperatures after both moderate and severe HIE is required. As the therapeutic range of hypothermia is thought to be 2–6 °C below normothermia^5^, a critical question is whether *more* cooling is beneficial once within the therapeutic range. Importantly, very few preclinical studies have directly compared multiple temperatures of HT. Those that have, describe either equivalent^6^,^7^ or contrasting effects^8^. In the Vannucci model (also known as the Levine or Rice-Vannucci model) of unilateral hypoxia-ischaemia (HI) in neonatal rats^9^, a well-validated model in the field of neonatal HIE, convention describes normothermia as 37 °C^9^,^10^,^11^,^12^. In this model, HT has been reliably shown to provide robust neuroprotection, with most studies cooling by 5 °C to a target of 32 °C^10^,^14^,^15^,^16^. As neonates are only cooled by 3.5 °C (from a normothermia of 37 °C), a relevant clinical question would be whether 3.5 °C, 5 °C, or deeper cooling provides greater neuroprotection.

The efficacy of HT after severe HIE is also still disputed. In subgroup analyses of infants with severe encephalopathy, one meta-analysis found an overall benefit of HT^1^ while another did not^17^. A failure of standard HT temperatures to provide neuroprotection after severe injury has been seen in both piglet^18^, and rat models of HIE^19^,^20^, though a broader range of potential treatment temperatures has yet to be investigated after severe HI. The current study aims to address some of these knowledge gaps by comparing the neuroprotective effect of serial temperatures within the therapeutic range of HT, as well as deeper cooling, in both moderate and severe models of HI brain injury.

## Results

### Mortality and exclusions

A total of 430 postnatal-day seven (P7) rat pups were used. In the moderate and severe HI models, 280 and 150 rat pups were used, respectively. In the HI experiments, 84 animals were excluded from the analyses due to death during surgical left common carotid artery ligation (n = 8, 1.9%) or during hypoxia (n = 14, 3.3%), failure to gain weight (n = 3, 0.7%), or because they carried temperature probes (n = 58, 13.5%). Twenty-one animals were used for control temperature measurements (n = 451 total).

### Baseline characteristics and weight gain

There were no differences in sex, weight at P7, or anaesthesia time during carotid artery ligation between groups. In both the moderate and severe models, one-way ANOVA showed an effect of treatment temperature on weight gain (moderate: p = 0.011, F = 3.028; severe: p = 0.004, F = 3.611). Tukey’s *post-hoc* test found that the HT18 group gained significantly less weight than the NT, HT33.5, and HT32 groups in the moderate model. In the severe model, the HT18 group gained significantly less weight than the HT33.5 group. No other between-group differences were found (Table 1).

### Hypothermic neuroprotection after moderate HIE follows a U-shaped curve

**Figure 1:**
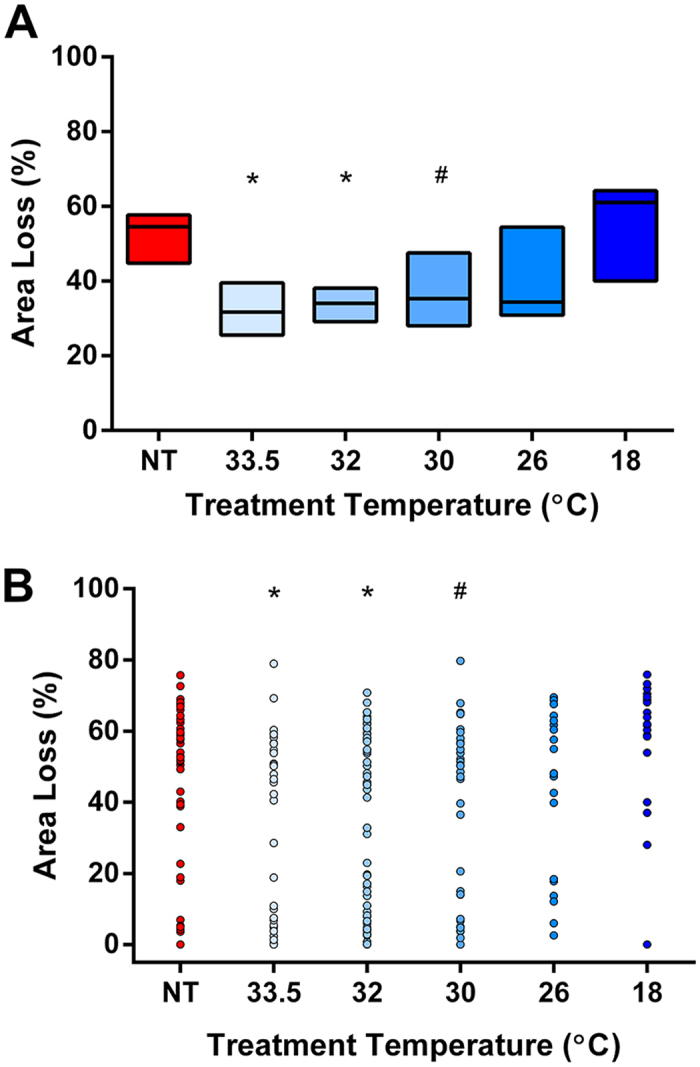
Hemispheric area loss after moderate hypoxia-ischaemia. Box plot (Hodges-Lehmann median with 95% CI) (**A**) and scatter plot (**B**) of hemispheric area loss across the six treatment groups. Compared to NT, significant neuroprotection was seen in the HT33.5 (p = 0.001), HT32 (p = 0.001), and HT30 (p = 0.01) groups. *Denotes p = 0.001 compared to NT control group. ^#^Denotes p = 0.01 compared to NT control group.

Across the hypothermia treatment groups, a temperature-dependent U-shaped curve of neuroprotection was seen (Fig. 1). Median hemispheric area loss of the left side was 54.6% in the NT group, with around 40% neuroprotection in the HT33.5, HT32, and HT30 groups (Table 2). Comparison of the 95% confidence intervals of the median indicated that there was no difference in area loss between those groups. No evidence of neuroprotection was seen in the HT26 group. A trend towards increased damage was seen in the HT18 group (p = 0.057). A representative coronal section and calculation of percent area loss is shown in Supplementary Figure S1.

### Hippocampal loss correlates with hemispheric area loss after moderate HIE

**Figure 2:**
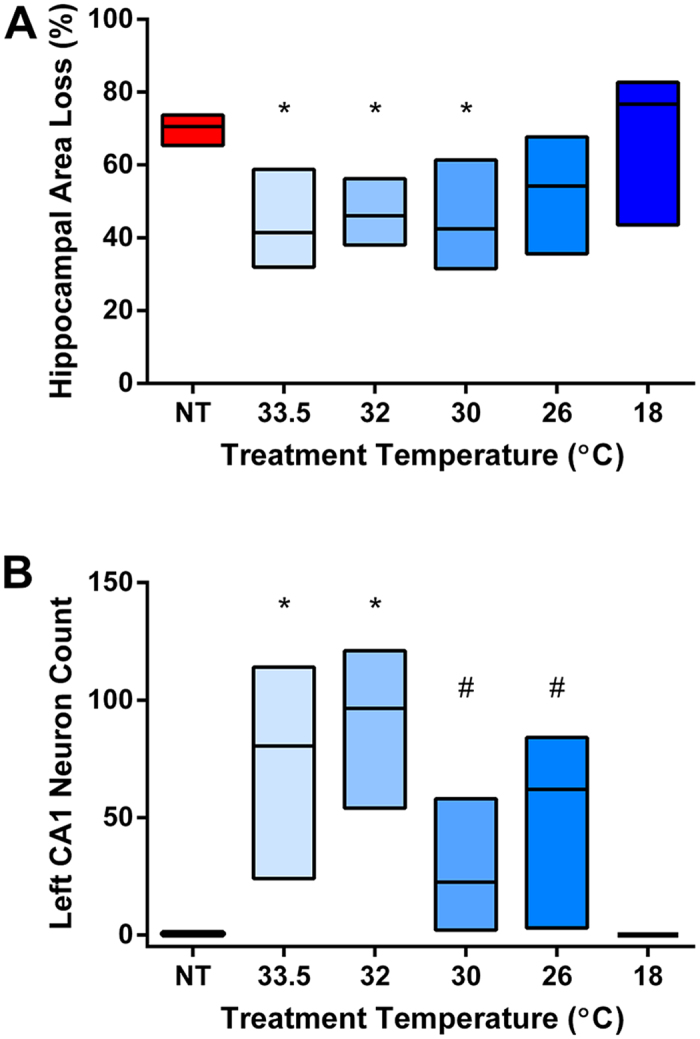
Hippocampal neuropathology after moderate hypoxia-ischaemia. Box plots showing Hodges-Lehmann median with 95% CI. (**A**) Hippocampal area loss across the six treatment groups. *denotes significant difference compared to NT control group. A significant reduction in hippocampal area loss was seen in the HT33.5 (p = 0.001), HT32 (p = 0.003), and HT30 (p = 0.003) groups. (**B**) Total left hippocampal CA1 pyramidal neuron count summed across three ROIs. Significant protection of CA1 pyramidal neurons was seen in the HT33.5 (p < 0.0001), HT32 (p < 0.0001), HT30 (p = 0.007), and HT26 (p = 0.008) groups. *Denotes highly significant difference compared to NT (p < 0.0001). ^#^Denotes significant difference compared to NT (p < 0.01).

Hippocampal area loss measurements across the treatment groups are shown in Table 2. As with hemispheric area loss, around 40% neuroprotection of hippocampal area was seen in the HT33.5, HT32, and HT30 groups (Fig. 2A). Of the 236 animals used for hemispheric area loss measurements, 19 (NT, n = 4; HT33.5, n = 3; HT32, n = 4; HT30, n = 2; HT26, n = 2; HT18 n = 4) could not be used for hippocampal loss analysis due to oblique sectioning or physical damage to the hippocampal area during processing. Linear regression analysis showed a highly significant correlation between hemispheric loss and hippocampal area loss (R2 = 0.855, p < 0.0001), with hippocampal loss tending to be greater (B = 1.209) than hemispheric area loss. Comparison of the confidence intervals indicated that there was no difference in hippocampal loss between groups (Table 2). No neuroprotection was seen in the HT26 or HT18 groups.

### Cooler isn’t better for hippocampal CA1 pyramidal neurons

**Figure 3:**
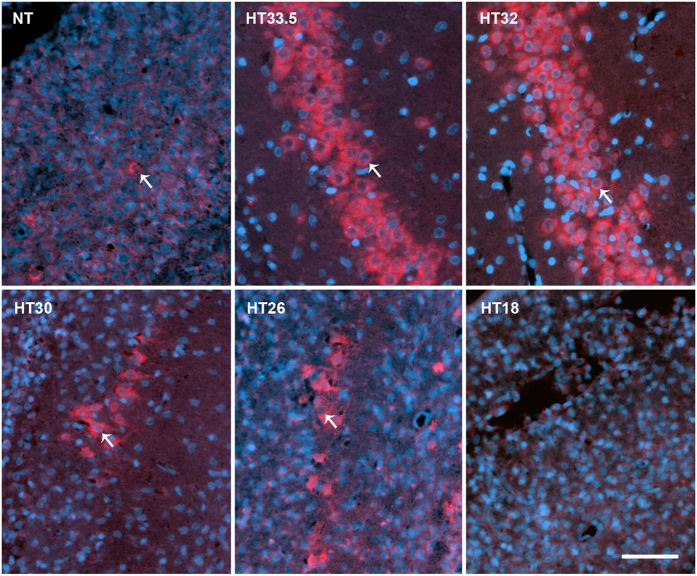
Hippocampal immunohistochemistry. Representative CA1 hippocampal regions of interest from each treatment group (indicated in the top left hand side of each image). Arrows show typical viable pyramidal neurons with large, round nuclei (DAPI, blue), and NeuN co-staining (red). Scale bar (bottom right) represents 50 μm.

A subset of animals was used to determine the effect of different treatment temperatures on hippocampal CA1 pyramidal neuron counts. Three animals (5%) could not be included due to damage to the sections during processing. Significant protection of ipsilateral CA1 pyramidal neurons was seen in the HT33.5, HT32, HT30, and HT26 groups (Fig. 2B). Within the therapeutic range of HT, there was a trend towards greater neuroprotection of pyramidal neurons in the HT33.5 and HT32 groups compared to the HT30 group (Table 2). Representative regions of interest (ROIs) from each treatment group are shown in Fig. 3. Total CA1 neuron count on the right (contralateral) side of the brain was similar across all six groups, with no effect of treatment temperature or ipsilateral injury severity (Supplementary Figure S2). Representative hippocampal ROIs from control P14 animals are shown in Supplementary Figure S3.

### Hypothermia at any temperature is not neuroprotective after severe HIE

**Figure 4:**
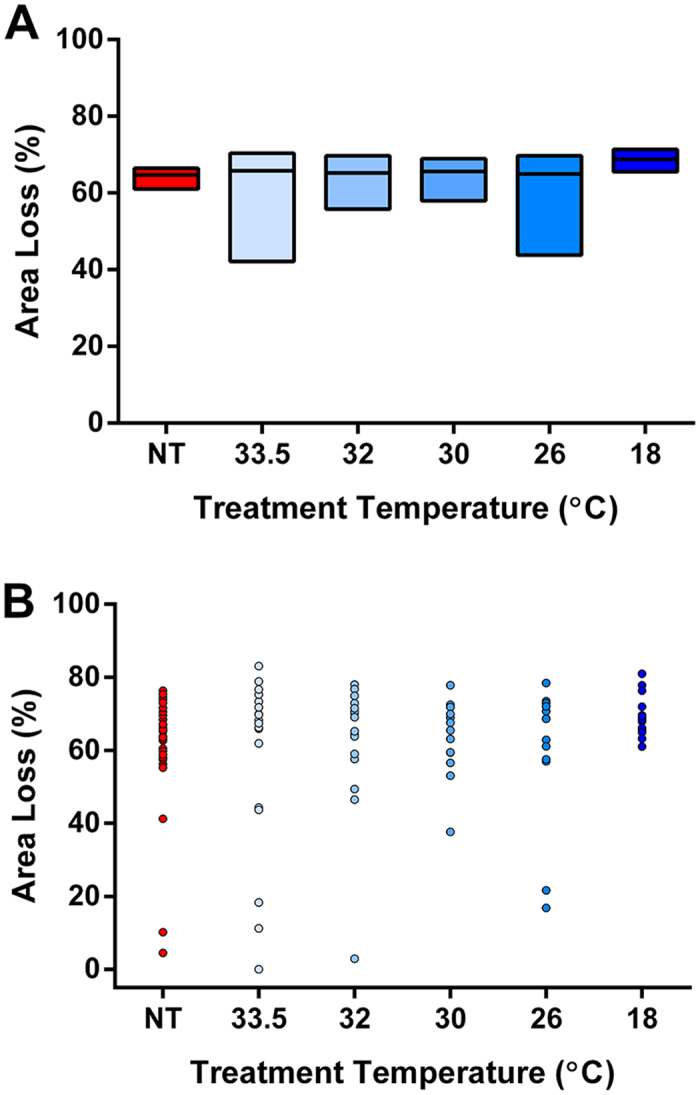
Hemispheric area loss after severe hypoxia-ischaemia. Box plot (Hodges-Lehmann median with 95% CI) (**A**) and scatter plot (**B**) of hemispheric area loss across the six treatment groups in the severe model. The HT18 group showed a trend towards greater area loss (p = 0.035) compared to NT control group.

After severe HI, in contrast to moderate HI, no evidence of neuroprotection was seen at any temperature evaluated in this study (Fig. 4). Comparison of the confidence intervals across the five different HT groups indicated that there was no difference in hippocampal loss between those groups. Compared to NT (37 °C), deep HT treatment (18 °C) resulted in a trend towards greater ipsilateral hemispheric area loss (p = 0.035).

### Observed core temperature in healthy developing rat pups

**Figure 5:**
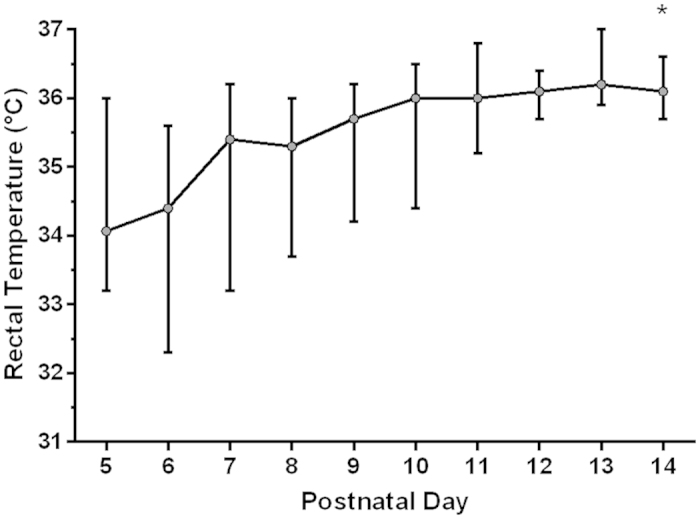
Temperature development in healthy laboratory rat pups. Graph shows median rectal temperatures (with range) in control pups from P5 to P14. Over the second week of life, median rectal temperature increased, while temperature variability decreased. *denotes significant increase in temperature compared to P7 (p < 0.0001).

In order to establish a normothermia range for developing neonatal rat pups in our experimental laboratory setting, serial rectal temperature measurements were taken from nesting healthy pups between P5 and P14 (Fig. 5). Pups were housed with their dam in an environmental temperature maintained at 21 °C. At P5, median rectal temperature was 33.9 °C (range 33.2–36.0 °C, n = 21). Median rectal temperature increased significantly from 35.4 °C (range 33.2–36.2 °C, n = 21) at P7 to 36.0 °C at P10 (34.4–36.5, p = 0.001; n = 20), and 36.1 °C at P14 (35.7–36.6 °C, p < 0.0001; n = 19).

## Discussion

For asphyxiated infants with mild or moderate HIE, therapeutic hypothermia is the current standard of care^2^. These recommendations are based on the first trials of HT for HIE, which showed improved outcomes at 18 months^21^,^22^,^23^ that persist into childhood^24^. Although HT is currently the only validated treatment for perinatal asphyxia, it is not universally neuroprotective. Unfortunately, 40–50% of treated infants with HIE will still die or have significant neurological disability^17^. As the development of HT into a neuroprotective strategy for neonates was based on preclinical research in a number of animal models^25^, there is scope to use these same models to further refine and optimise cooling protocols. For instance, early results showing the neuroprotective effects of HT in the Vannucci rat model were accurately replicated in larger animal models (piglets and fetal sheep), before being translated into the first pilot and clinical trials in neonates^25^. However, many fundamental questions, including what temperature should be considered normothermia in the control group, and how much active cooling is required for optimal neuroprotection, remain unanswered. The data presented here show that in a model of moderate HI injury, there is a distinct temperature-dependent neuroprotective effect of HT. Conversely, in the model of severe injury, HT does not provide neuroprotection, regardless of treatment temperature. Serial temperature measurements from nesting laboratory rat pups also suggest that normothermia at P7 is 1–2 °C below the temperatures historically used in control “normothermia” groups in the Vannucci model.

As a result of work in a number of animal models, the therapeutic range of HT is thought to be 2–6 °C below normothermia^5^. In fetal sheep, a reduction in extradural temperature by more than 5.5 °C, and oesophageal temperature by around 2 °C, was required to see significant neuroprotection after 30 min of bilateral carotid artery occlusion^26^. In piglet models of HIE, whole-body HT to achieve reductions in core temperature by 2.5 °C^27^, 4 °C^18^ and 5 °C^28^ have all produced significant neuroprotection. More recently, Alonso-Alconada *et al*. found that 3.5 °C or 5 °C of cooling provides equivalent neuroprotection in piglets, with increased damage at lower temperatures (8.5 °C cooling)^29^. In line with this work, we describe a U-shaped curve of hypothermic neuroprotection after moderate HI. With hemispheric area loss, significant neuroprotection was seen in the 33.5 °C, 32 °C, and 30 °C groups, with no protection below 30 °C. Compared to hemispheric loss, greater hippocampal loss was seen across all treatment groups. Milder HT in the 33.5 °C group was not significantly different compared to the 32 °C group, and a trend towards greater hippocampal neuroprotection was seen in these two groups compared to 30 °C. Cooling to 26 °C conferred some neuroprotection of hippocampal CA1 pyramidal neurons, which are particularly susceptible to injury in this model^30^. Different temperatures may therefore be more beneficial for different sub-structures within the brain, and this appears to be particularly evident in larger animal models. For example, Iwata *et al*. compared induced hypothermia of 3.5 °C and 5.5 °C (from a normothermia of 38.5 °C) in a piglet bilateral carotid artery occlusion model of HIE. Cooling by 5.5 °C resulted in a greater reduction in neuronal death in the cortical grey matter, but in the deep grey matter, greater neuroprotection was seen in the 3.5 °C group^8^. For this reason especially, ongoing work in animals such as piglets or fetal sheep will be important in order to ascertain whether current cooling protocols can be optimised further.

After moderate HI in the current study, very deep cooling to 18 °C did not provide any neuroprotection. Overcooling is therefore likely to result in a loss of benefit, and a potential trend towards harm. Indeed, animals that were cooled to 18 °C for 5 h after HI gained significantly less weight compared to other groups after both severe and moderate HIE, which indicates a temperature-dependent neurological injury that impaired ability to feed. Detrimental effects of very deep cooling have also been described in an adult dog model of cardiac arrest-induced brain injury^31^. Due to the longer rewarming period, pups in the HT30, HT26, and HT18 groups also spent more time away from their littermates and dam. However, we have previously shown that 10 h of cooling does not worsen injury compared to 5 h^20^, therefore it is unlikely that the extended time away from the litter in these groups will have affected the extent of injury.

One issue with determining the appropriate depth of cooling after HIE is the need for a defined “normothermia” in the control group, which will depend on both the age and relative maturity of the animal model being used. In our previous work with the Vannucci model in the P7 rat pup, we have repeatedly found a significant neuroprotective effect of cooling to 32 °C for 5 h immediately after hypoxia^10^,^14^,^20^. This is compared to control rats maintained at 37 °C. Though 37 °C lies within the range of adult rat normothermia^13^,^32^ the neonatal rat is largely poikilothermic at birth, and the first three weeks are critical to the development of thermal homeostasis^12^,^13^,^33^,^34^. Therefore, “normothermia” for P7-P14 rats is likely to be highly variable, depending on both age and ambient temperature^13^,^32^. Accordingly, we found that median rectal temperature rose from 35.4 °C to 36.1 °C, and became less variable, between P7 and P14. Position in the nest, huddling^35^, distance from the dam, and length of time since last period of suckling, would likely explain the larger temperature variability seen in younger pups. Importantly, no animal had a core temperature reading of 37 °C or above at any age up to P14. Physiological normothermia for a P7 rat pup, bred and housed under modern laboratory conditions, is therefore likely to lie between 33.5 °C and 36 °C. Even in huddles, core temperature rapidly drops when rat pups are without the dam and exposed to environmental temperatures similar to that of a modern laboratory^35^. This is almost certainly a normal physiological occurrence that the developing rat is adapted to. This wide range of “normothermia” in the P7 rat represents a clear limitation when attempting to draw any conclusion regarding optimal treatment temperature from this data alone. However, we do not have any reason to believe that our nesting temperatures are abnormally variable. To our knowledge, no robust baseline laboratory data of nesting temperatures from animals has ever been formally published by groups investigating the Vannucci model. One recent publication reported mean nesting temperature in P7 rats to be 36.6 °C, but the range (minimum and maximum temperature) and ambient temperature was not given^36^. Two recent publications from another group show mean temperatures in P7 rats of 33.0 °C–35.5 °C before undergoing HI^37^,^38^. This variability is likely to be a limitation of the model, and extraction of any single optimal cooling temperature will require in-depth knowledge of the local housing conditions and baseline normothermia temperatures. As the P7 rat is thought to be slightly immature compared to the term newborn^39^, a term-equivalent Vannucci model has recently been developed in the P10 rat, with a temperature range of 36.5 °C–38.1 °C in the normothermia group (environmental temperature of 35.5 °C)^16^. In our study, median nesting temperature of P10 rats was 36.0 °C, which suggests that normothermia of the control group should be individualised to the maturity of the animal, as well as the local experimental set-up.

Clinical trials of HT in asphyxiated neonates may have had similar issues regarding normothermia in the control groups. In the initial trials of whole-body HT (cooling to 33.5 °C for 72 h) after HIE, target mean core (oesophageal or rectal) temperature in the control groups was also maintained around 37 °C^21^,^23^. However, normothermia for healthy term babies is below 36.1 °C (97 °F), slowly increasing over the first 24 hours as the baby adjusts to the extra-uterine environment^40^. In moderately-asphyxiated self-ventilating neonates, temperature spontaneously drops to as low as 34.4 °C (94 °F), and recovers more slowly^40^. Historical data therefore suggests that rapidly heating asphyxiated newborns with overhead heaters and maintaining the control groups at 37 °C^23^ is likely to have increased temperature above physiological normothermia. In addition, as fever is known to worsen neurological outcome after HI in animal models^41^, adults^42^, and neonates^43^, evidence is mounting to support the idea that prevention of hyperthermia may be as important as active hypothermia after cerebral HI. This was the conclusion of the Targeted Temperature management (TTM) trial in adults after cardiac arrest, where 24 h of cooling to 33 °C or strict maintenance of 36 °C provided equivalent neuroprotection^44^. As physiological normothermia for the term neonate appears to be below 37 °C, it is possible that the robust long-term benefits of HT seen in clinical trials of asphyxiated term newborns could be due to the induction of a relative hyperthermia (37 °C, by management under an overhead heater) in the control groups, with an associated worsening of outcome compared to the HT groups.

With the current data, we show greater neuroprotection from cooling by 2–3.5 °C (from a median normothermia of 35.4 °C) than cooling by 5.4 °C, and that the optimal treatment temperature likely lies somewhere between 32 °C and 33.5 °C. As hypothermia after neonatal HIE acts through multiple pathways across multiple temporal phases^45^,^46^, different temperatures may exert a varying balance of metabolic, immunomodulatory, and neurotrophic effects. Importantly, there is little evidence that cooling by more than 3.5 °C (below 32 °C) would provide additional benefit. Though the mechanisms of hypothermic neuroprotection are wide-ranging, they are still poorly understood. Importantly, certain assumed neuroprotective effects of TH, such as suppression of cytotoxic oedema and prevention of excitatory amino acid accumulation are not as robustly supported by the experimental evidence as previously thought^46^. Hypothermia within the therapeutic range exerts a profound anti-apoptotic effect, as well as preventing secondary increases in neuroinflammation following HI brain injury^45^,^46^. However, as metabolism decreases linearly with core temperature, all cellular metabolic processes will be affected by deeper HT^47^. As well as suppressing progression of injury, this may also affect beneficial adaptations and responses to HI that could otherwise promote recovery. Two potential pathways that may be affected by deeper cooling, and could therefore benefit from HT at the milder end of the therapeutic range, are induction of hypoxia-inducible factor-1α (HIF-1α), and free radical production. HIF-1α is increased in the hours following a period of hypoxia, resulting in upregulation of genes associated with erythropoiesis and angiogenesis, and altered balance of cell death pathways^48^. HIF-1α, as part of the transcription factor HIF-1, increases erythropoietin (EPO) expression. EPO has been shown to have multiple neuroprotective effects in models of neonatal HIE^49^. However, the role of HIF-1α in neuroprotection is complex. Both inhibition and increased production of HIF-1α have been associated with reduced apoptosis after HI brain injury^50^, with the exact outcome probably depending on both level and timing of HIF-1α activation. While prolonged hypothermia can suppress HIF-1 activation^51^, xenon gas, which provides additive neuroprotection in combination with TH, may increase HIF-1α as part of its neuroprotective effect^14^,^52^. Reducing a TH-induced suppression of HIF-1α activation is one potential benefit of milder TH.

Another pathway that may affect recovery is free radical production, increases in which are associated with greater neurological injury after HI^53^. Physiological increases in free radicals such as reactive oxygen species (ROS) are also associated with increased mitochondrial function and improved cellular antioxidant status, at least in part through activation of nuclear factor (erythroid-2)-related factor 2 (Nrf2), which has been shown to be involved in the mechanism of multiple neuroprotective strategies^54^,^55^,^56^,^57^. While HT is generally thought to suppress production of ROS, deeper HT may increase ROS production, as well as inhibit ROS clearance due to slower enzyme kinetics^45^,^54^,^58^. Hypothermia at the lower end of the therapeutic range may therefore negatively affect the balance of ROS production and subsequent adaptation. In cultured microglia, mild HT treatment at 35 °C also suppresses production of pro-inflammatory cytokines that reduce cell viability after stimulation with toll-like receptor (TLR) agonists^59^. Though the inflammation-suppressing effect was smaller than that seen at 33 °C, treatment at 35 °C may be sufficient to provide neuroprotection without risking the potential negative physiological effects of deeper cooling^47^,^59^. The balance of anti-apoptotic and anti-inflammatory effects of HT versus maintenance of positive adaptations after injury, and their temperature-dependence, is a crucial area for future research in models of HIE.

One potential limitation of this work is the short-term (one week) survival compared to the clinical setting, where the goal is long-term improvements in neurological outcome. In the Vannucci model, certain short-term neuropathological markers do not always predict long-term injury^60^, and behavioural improvements after neuroprotective treatment can be seen that are not predicted purely by pathology^61^. However, we have previously randomised animals to either short-term (P14) or long-term (P49) survival and showed that global pathology score across the basal ganglia, cortex, hippocampus, and thalamic regions, and response to TH, follows a similar pattern at both time points^10^. Pathology score and area loss are also directly correlated with one-another, as well as with sensorimotor function at P42 and P49^10^,^20^. This suggests that we can use these short-term markers to identify useful temperature comparisons for more in-depth future investigations into mechanism and outcome. As we previously found that the hippocampus is the area that is most sensitive to the neuroprotective effects of HT at P14^10^,^15^, we also employed hippocampal area loss and CA1 neuronal counts in order to ascertain any further temperature-dependent changes in the hippocampus at this early time point^62^,^63^. However, in order to truly investigate whether milder cooling provides equivalent neuroprotection, long-term improvements in neurobehavioural outcome should be demonstrated, and this will be a focus of future work. Overall, these data in the neonatal rat, as well as recent work in the piglet^29^ and in the fetal sheep^64^, suggest that longer and or deeper cooling does not provide additional benefit after neonatal HIE. In fact, deeper cooling may be associated with increased neurological injury. This mirrors the results from the recent “longer and deeper” trial in humans, which was stopped early due to a lack of benefit in the longer (120 h vs 72 h) and cooler (32 °C vs 33.5 °C) groups^3^. It has since been discussed whether preclinical studies could have directly investigated the issue of longer or deeper cooling before an exploratory trial in human neonates was initiated^25^.

In agreement with our previous results^20^, HT did not provide neuroprotection after severe HIE. The more severely-damaged brain may in fact be at greater risk of the deleterious effects of accidental over-cooling during HT, as the group cooled to 18 °C in the severe HI model showed a trend towards increased damage compared to the control group. As we examined two models of severity, with six treatment groups in each, a large number of animals were used. Due to the well documented high degree of variability in the moderate model^16^, NT and HT32 groups were included in all experiments in order to ensure reproducibility of results, which increased numbers in those groups. In the severe model, all experiments indicated a lack of effect of HT, and increasing group sizes even further would have been unethical. Future clinical work should focus on the stratification of injury severity, with treatment adjusted accordingly. With further investigation, it may transpire that infants with severe encephalopathy would derive greater benefit from treatments other than HT, such as erythropoietin, melatonin, xenon, or umbilical cord blood cells, which are all currently being investigated in early clinical trials^49^,^65^,^66^,^67^.

No differences in pathology were seen between males and females at any temperature in either the moderate or the severe model. This does not preclude sex-dependent differences in outcome that may also differ based on the treatment temperature. Sexually dimorphic responses to perinatal or neonatal brain injury and subsequent treatment have been seen in both preclinical models and the clinical setting^36^,^60^,^68^,^69^. However, the experiment was not designed to look for any differences between sexes, and these results are also in line with what we have previously seen in our model^15^,^20^,^61^. As well as the effects of injury severity and HT temperature on outcome, sex differences in response to TH, and potential adjustment of treatment protocols based on these differences and their underlying mechanisms, are an important area for future work.

In summary, we describe a temperature-dependent U-shaped curve of hypothermia neuroprotection after moderate HI. Serial temperature measurements in healthy rats, and historical data from healthy and asphyxiated neonates, suggests the previous preclinical and clinical work in these groups has involved active hyperthermia (warming to ≥37 °C) in the “normothermic” controls^40^. This may have increased the disparity between the control and HT treatment groups by increasing injury in the control group. We have also shown that greater neuroprotection is potentially seen at the milder end of the therapeutic range, and that deeper HT does not provide greater neuroprotection. As the Vannucci rat model and HIE models in larger mammals have a strong track-record of clinical translation for the development of hypothermia protocols for neonatal HIE, investigation into the optimal depth and duration of hypothermia should continue to be carried out in the laboratory in order to inform the design of future clinical trials. Based on these results, we recommend that future trials of hypothermia in neonates consider the use of milder whole-body cooling, or maintenance of normothermia and prevention of hyperthermia, in the range of 34 °C to 36 °C.

## Methods

### Animals

The effects of variable-depth hypothermia on neonatal hypoxic-ischaemic (HI) brain injury were assessed using a modified Vannucci rat-model of unilateral HI^70^. Post-natal day seven (P7) rat pups are considered to have an equivalent level of brain maturation to the 36 week-gestation human neonate^39^. All experimental protocols were reviewed and approved by the University of Oslo’s animal ethics research committee. The methods described below were carried out in accordance with those approved protocols, as well as the University of Oslo’s ethical guidelines regarding the use of experimental animals. Litters of Wistar rat pups (Charles River, Sulzfeld, Germany) of both sexes were kept in an animal facility with a 12:12 h dark:light cycle at 21 °C environmental temperature, with food and *water ad libitum*, and checked for health daily.

### Moderate Vannucci model of hypoxia-ischaemia

On P7, pups underwent ligation of the left carotid artery under anaesthesia, with 3% isoflurane in a 2:1 gas mixture of NO_2_/O_2,_ via a nose cone. Average anaesthesia time was kept under 6 minutes (Table 1) to minimise hypoxic pre-conditioning due to isoflurane-induced respiratory depression. After recovering under a heat lamp, pups were returned to the dams for at least 30 minutes, before being exposed to 8% oxygen for 100 minutes at 36 °C in a specially-designed chamber^14^. This length of hypoxia produces a “moderate” injury, with around 40% loss of the left hemisphere compared to the right^20^. As both ischaemia and superimposed hypoxia are required to induce brain injury in the neonatal rat, the right (unligated or contralateral) hemisphere remains undamaged, and can act as an internal control^70^,^71^. During hypoxia, core temperatures were continuously recorded in each chamber in “sentinel” pups carrying a rectal temperature probe (IT-21, Physitemp Instruments, Clifton, NJ, USA). Rectal temperature was maintained within ±0.2 °C of the target using a servo-controlled water-filled mat (CritiCool, MTRE, Yavne, Israel) inside the chamber. In P7 rats, rectal temperature correlates within 0.1 °C with brain temperature^71^.

### Severe Vannucci model of hypoxia-ischaemia

To assess the neuroprotective effects of varying treatment temperature after severe HI, a further-modified version of the Vannucci model was developed^20^. In this model, both the length of exposure to hypoxia and insult temperature were increased, to 150 minutes and 37 °C, respectively. This produces around 50% greater injury compared to the moderate model (60% loss of the left hemisphere compared to the right)^20^.

### Hypothermia treatment groups

Before hypoxia, pups were randomised by litter, weight, and sex to receive one of six different treatment temperatures for 5 h. In this model, normothermia (NT) refers to 37 °C, with standard hypothermia (HT) treatment occurring at 32 °C (HT32)^10^,^14^,^15^. To directly compare the effect of both milder and deeper cooling, four other treatment groups were designated to be cooled to 33.5 °C (HT33.5), 30 °C (HT30), 26 °C (HT26), or 18 °C (HT18). Cooling as low as 18 °C is commonly used as a prophylactic neuroprotective measure during neonatal cardiac surgery^72^. Immediately after hypoxia, pups were transferred to pre-cooled chambers at the allotted temperature. Rectal temperature was maintained within ±0.2 °C of the target, as described above. After 5 h of the allocated treatment, pups were removed from the treatment chambers and returned to the dams^20^. In groups cooled below 32 °C, pups were rewarmed at 1 °C every 15 minutes until they reached a temperature of 33–34 °C, before being returned to the dam. This was to prevent rapid rewarming due to handling and nesting, as rapid rewarming may negate some of the neuroprotective effects of HT after HIE^73^.

### Tissue harvesting and processing

At P14, rats were sacrificed via transcardiac perfusion with saline and 10% neutral-buffered formalin under isoflurane/N_2_O anaesthesia. Brains were harvested and kept in 10% neutral-buffered formalin for four days until further processing. Six coronal 3 mm slices were cut through the brain using a standard rat brain matrix (ASI Instruments Inc., Warren, MI, USA), and embedded in paraffin.

### Area loss analysis

Area loss analysis was performed as previously described^20^. The method is depicted in Supplementary Figure S1. Briefly, 5 μm sections from the slices best representing the cortex, hippocampus, basal ganglia and thalamus were taken, and stained with haematoxylin and eosin (H&E). Slides were scanned (Epson Perfection V750 Pro), and virtual slides were exported as 600dpi images. The optical density and hemispheric area of each section was analysed with ImageJ software (ImageJ, version 1.46r, National Institutes of Health, Bethesda, MD, USA) by an individual who was blinded to group allocation (Supplemental Figure 1). The average percentage area loss from four sections (two at the level of the frontal cortex, and two at the mid-hippocampal level) was calculated by using the following formula: (1−(area left/area right))×100. In this model, computer-assessed percent hemispheric area loss has previously been shown to be highly correlated with formal neuropathology assessment^20^.

### Hippocampal loss analysis

Hippocampal area loss was analysed in the moderate model, as previously described^62^,^63^. Sections at the level of the hippocampi were scanned at a higher resolution (2400dpi), and two consecutive sections from each animal were analysed with ImageJ. A subset of sections were examined for hemispheric and hippocampal areas by a second blinded assessor to check for inter-rater reliability^62^. In the severe model, hippocampal loss was not assessed due to extensive ipsilateral hippocampal loss in all animals.

### Hippocampal CA1 neuron assessment

To evaluate hippocampal neuron loss in a representative subset of animals from each treatment group in the moderate model, immunohistochemistry was used to count pyramidal neurons in the CA1 region of the hippocampus. This region is known to be particularly vulnerable to hypoxia at P7^30^. In each treatment group, further sections were taken from the 10 animals with area loss values spanning the median value for that treatment. Practically, this involved ranking all the animals in each treatment group by area loss, and selecting the middle 10 animals for CA1 neuron counting via immunohistochemistry.

### Immunohistochemistry

Paraffin-embedded tissue was deparaffinised in xylene and rehydrated in decreasing concentrations of ethanol. Antigen retrieval was performed in citrate buffer (pH 6.0), using a PT link instrument (Dako, Denmark). After blocking with 10% goat serum, a primary mouse antibody against NeuN (1:500; Millipore, MA, USA) was applied overnight at room temperature. Slices were rinsed with PBS and incubated for 1 h at room temperature with secondary Alexa Fluor 568 (Invitrogen, 1:500) antibodies. Slides were rinsed again, and coverslipped with ProLong Gold with 4′,6-diamidino-2-phenylindole (DAPI, Invitrogen). Sections were scanned with a virtual microscopy scanner (Axio Scan.Z1, Carl Zeiss, Jena, Germany) in fluorescence mode with a plan apochromatic 20x lens. Virtual slides were then exported as high-resolution tiff images. Three consecutive non-overlapping ROIs were assessed from the CA1 region of both hippocampi^74^. Cells located within the pyramidal layer, which were visible within the plane of focus, and positive for both NeuN (neuronal marker) and DAPI (cell nucleus), were counted as neurons. Healthy neurons were identified as those with large, round nuclei, and paler areas indicating unstained nucleoli. The total number of viable pyramidal neurons across the three ROIs from each hippocampus was then summed. Cell counting was performed by an observer blinded to the treatment group. A subset of regions (25%) was re-assessed by a second blinded observer, and inter-observer reliability was determined to ensure reproducibility.

### Core temperature in developing neonatal rat pups

In order to establish the normothermic range in developing rat pups in an experimental setting, rectal temperature in healthy pups was measured daily from P5 to P14. Two pregnant Wistar dams were obtained at gestation day 18. Every day, from P5 onwards, pups were individually removed from the nest at random. Rectal temperature for each rat pup was assessed immediately after removal from the nest to minimise heat losses to the environment, using the same temperature probes as used during hypoxia. The pups were then weighed, and returned to the dam.

### Statistical analysis

Pups used as rectal and skin temperature probes were excluded from the final analysis because the stress of restraint at normothermia has previously been shown to have a neuroprotective effect in this model^75^. Statistical analyses were performed using SPSS software version 22 (SPSS Inc., Chicago, IL, USA). Baseline weight, anaesthesia time, and sex data were compared across groups using one-way ANOVA. A p-value < 0.05 was considered statistically significant. For area loss and hippocampal loss data that was not normally distributed, a Hodges-Lehmann median with 95% confidence interval (CI) was calculated (StatXact version 10; Cytel, Cambridge, MA, USA), as previously described^15^. To minimise issues regarding multiple comparisons across the six treatment groups, a maximum of five between-group comparisons of dependent variables were considered to be most relevant, and these were made subject to formal statistical testing. These comparisons were carried out with a two-sided Wilcoxon-Mann-Whitney two-sample test, using a Bonferroni correction. Therefore, for multiple comparisons across treatment groups, a p-value < 0.05/5 = 0.01 was considered to be statistically significant. For other dependent variables, the median and 95% confidence intervals are given.

## Additional Information

**How to cite this article**: Wood, T. *et al*. Treatment temperature and insult severity influence the neuroprotective effects of therapeutic hypothermia. *Sci. Rep.* **6**, 23430; doi: 10.1038/srep23430 (2016).

## Supplementary Material

Supplementary Information

## Figures and Tables

**Table 1:** Baseline characteristics and weight gain in the moderate and severe models.

Model	Group	N =	Male	Weight P7 (g)	Weight P14 (g)	Weight Gain (g)	Weight gain (%)	Anaesthesia time (min:sec)
Moderate	NT	72	32 (44.4%)	12.4 (1.7)	23.4 (3.7)	11.1 (2.8)	89.9 (21.4)	5:33 (1:13)
	HT33.5	39	20 (51.3%)	11.9 (1.5)	23.2 (2.8)	11.3 (2.7)	97.2 (28.3)	5:45 (1:27)
	HT32	73	36 (49.3%)	12.1 (1.6)	23.5 (3.6)	11.3 (2.7)	93.8 (22.1)	5:34 (1:17)
	HT30	38	20 (52.6%)	12.9 (1.7)	23.8 (4.0)	10.9 (2.9)	88.7 (20.8)	5:35 (1:15)
	HT26	24	12 (50.0%)	12.2 (1.5)	22.8 (3.2)	10.6 (2.5)	87.0 (20.7)	4:55 (0:50)
	HT18	25	13 (52.0%	12.2 (1.3)	21.3 (2.9)	9.1 (2.4)*	74.9 (19.2)	5:17 (0:59)
Severe	NT	40	18 (45.0%)	11.1 (1.6)	20.0 (3.1)	8.9 (2.5)	81.0 (24.6)	05:29 (1:32)
	HT33.5	22	11 (50.0%)	11.3 (1.4)	21.2 (4.0)	9.8 (3.3)	87.2 (28.0)	05:11 (1:37)
	HT32	20	11 (55.0%)	11.4 (1.5)	20.5 (3.3)	9.1 (2.5)	80.8 (21.1)	05:24 (1:24)
	HT30	19	6 (31.6%)	11.4 (1.5)	19.1 (2.6)	7.6 (1.9)	67.8 (18.9)	05:00 (1:29)
	HT26	14	7 (50.0%)	11.8 (1.6)	20.0 (3.2)	8.3 (2.6)	71.0 (24.4)	05:13 (0:54)
	HT18	17	9 (52.9%)	11.6 (1.2)	18.4 (1.3)	6.9 (1.4)^#^	60.5 (15.0)	05:39 (0:52)

Variables are shown as mean (standard deviation). *Significant compared to moderate NT, HT33.5, HT32, and HT30 groups. ^#^Significant compared to severe HT33.5 group.

**Table 2:** U-shaped hypothermic neuroprotection in the moderate model.

**Treatment Group**	Hemispheric Area Loss (%)		Hippocampal Area Loss (%)		Total Left CA1 neuron count	
	**N =**	**Median (95% CI)**	**N =**	**Median (95% CI)**	**N =**	**Median (95% CI)**
NT	62	54.6 (44.8–57.7)	58	70.5 (65.4–73.7)	10	1 (0–1)
HT33.5	35	31.7 (25.5–39.6)*	32	41.4 (31.2–58.8)*	9	80.5 (24–114)*
HT32	64	34.0 (29.2–38.2)*	60	46.0 (38.0–56.2)*	10	96.5 (54–121)*
HT30	34	35.3 (28.0–47.6)*	32	42.5 (31.6–61.4)*	8	22.5 (2–58)*
HT26	20	43.4 (30.9–54.5)	18	54.2 (34.6–67.7)	10	62 (3–84)*
HT18	21	61.0 (40.0–64.2)	17	76.7 (43.6–82.7)	10	0 (0–0)

Table shows hemispheric area loss, hippocampal area loss, and left CA1 hippocampal pyramidal neuron count in the moderate model, across six treatment groups. Data displayed as Hodges-Lehmann median with 95% confidence interval (CI). *Denotes significant difference compared to control (NT) group.
