# Supplementary material for: Treatment temperature and insult severity influence the neuroprotective effects of therapeutic hypothermia
Source: Sci Rep. 2016 Mar 21;6:23430. doi: 10.1038/srep23430 (PMC4800445; doi:10.1038/srep23430)
Supplement: Supplementary Information [file srep23430-s1.pdf]

Treatment temperature and insult severity influence the neuroprotective effects of therapeutic hypothermia

Supplementary Information

Thomas Wood, Damjan Osredkar, Maja Puchades, Elke Maes, Mari Falck, Torun Flatebø, Lars Walløe, Hemmen Sabir, Marianne Thoresen

Supplementary Figures

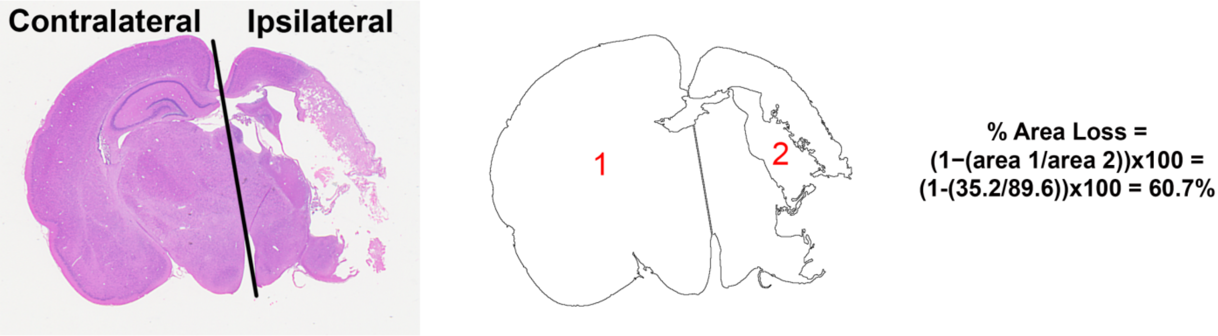

Supplementary Figure S1. Representative coronal section and calculation of percent area loss. A representative H&E stained coronal section (left) shows the damaged ipsilateral side, with uninjured contralateral side. Images are split in ImageJ (centre), and percent area loss calculated with the equation shown (right).

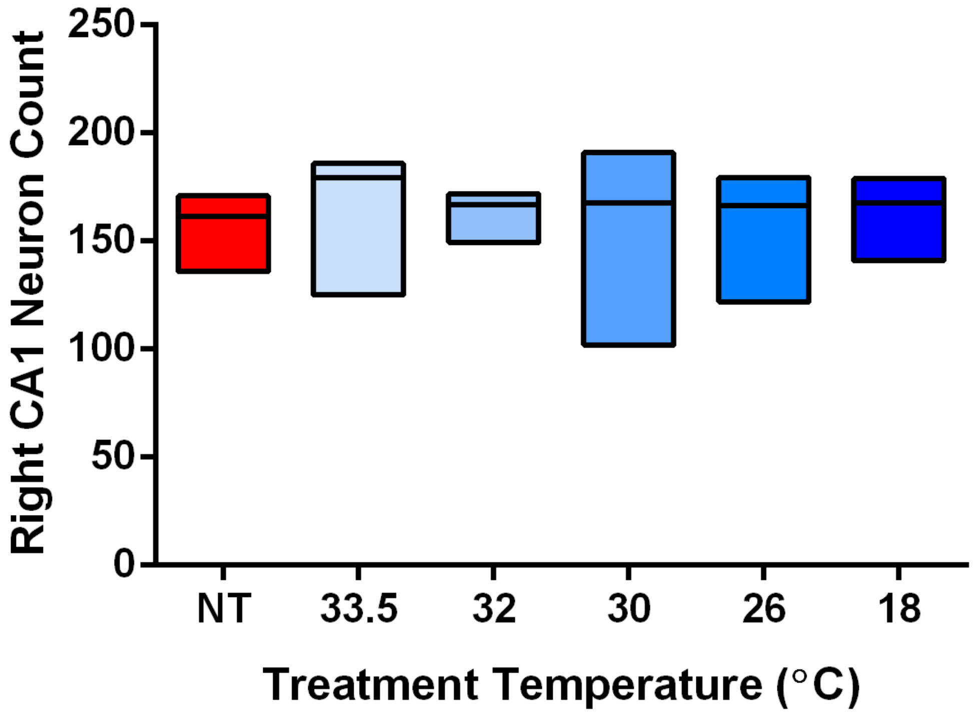

Supplementary Figure S2. Contralateral CA1 pyramidal neuron counts. Box plot of total right hippocampal CA1 pyramidal neuron count, summed across three ROIs, in the moderate model. Data shown as Hodges-Lehmann median with 95% CI. Median neuron count was 162 (136-171, n=10) in the NT group, 180 (125-186, n=9) in the HT33.5 group, 167 (150-172, n=10) in the HT32 group, 168 (102-191, n=10) in the HT30 group, 167 (122-180, n=10) in the HT 26 group, and 186 (141-179, n=10) in the HT18 group. No difference was seen across the treatment groups.

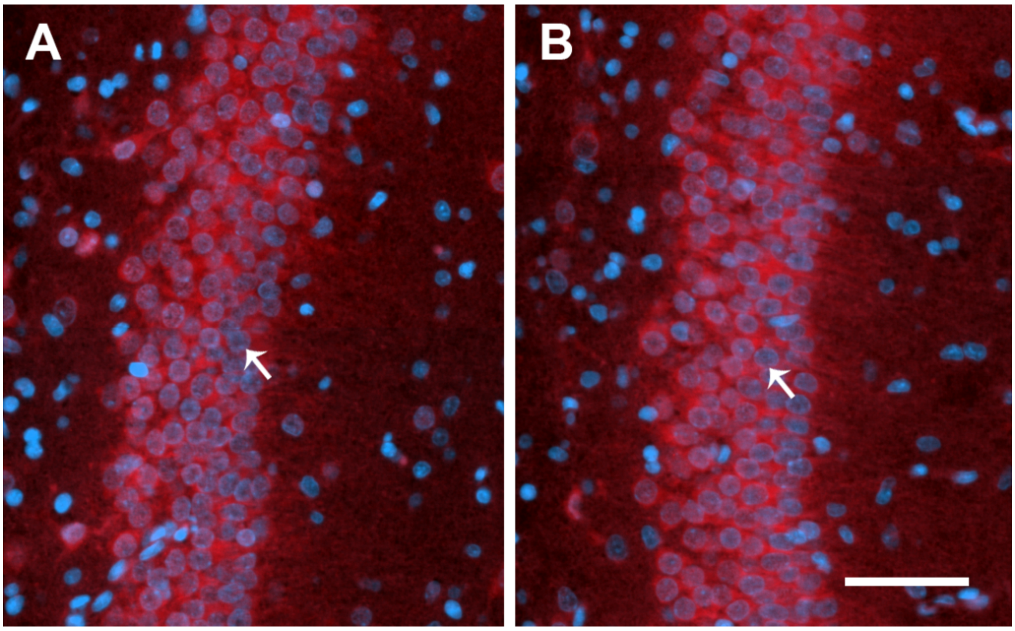

Supplementary Figure S3. Hippocampal immunohistochemsity. Representative CA1 hippocampal regions of interest from the left **(A)** and right **(B)** hippocampus of a non-ischaemic juvenile control animal at P14. Arrows show typical viable pyramidal neurons with large, round nuclei (DAPI, blue), and NeuN co-staining (red). Scale bar (bottom right) represents 50 µm.
